# Emergent Momentum-Space Skyrmion Texture on the Surface of Topological Insulators

**DOI:** 10.1038/srep45664

**Published:** 2017-04-05

**Authors:** Narayan Mohanta, Arno P. Kampf, Thilo Kopp

**Affiliations:** 1 Center for Electronic Correlations and Magnetism, Theoretical Physics III, Institute of Physics, University of Augsburg, 86135 Augsburg, Germany; 2 Center for Electronic Correlations and Magnetism, Experimental Physics VI, Institute of Physics, University of Augsburg, 86135 Augsburg, Germany

## Abstract

The quantum anomalous Hall effect has been theoretically predicted and experimentally verified in magnetic topological insulators. In addition, the surface states of these materials exhibit a hedgehoglike “spin” texture in momentum space. Here, we apply the previously formulated low-energy model for Bi_2_Se_3_, a parent compound for magnetic topological insulators, to a slab geometry in which an exchange field acts only within one of the surface layers. In this sample set up, the hedgehog transforms into a skyrmion texture beyond a critical exchange field. This critical field marks a transition between two topologically distinct phases. The topological phase transition takes place without energy gap closing at the Fermi level and leaves the transverse Hall conductance unchanged and quantized to *e*^2^/2*h*. The momentum-space skyrmion texture persists in a finite field range. It may find its realization in hybrid heterostructures with an interface between a three-dimensional topological insulator and a ferromagnetic insulator.

Breaking of time-reversal symmetry (TRS) in three-dimensional (3D) topological insulators (TIs)^1^,^2^,^3^,^4^ has led to fascinating new topological phenomena. Among them are the quantum anomalous Hall effect (QAHE)^5^,^6^,^7^,^8^,^9^,^10^, the inverse spin-galvanic effect^11^, axion electrodynamics^12^,^13^, and the half-quantum Hall effect on the surface with conductance *σ*_*xy*_ = *e*^2^/2*h*^14^. In TIs, strong spin-orbit coupling locks the electron’s spin to its momentum and forces the surface states to form a helical spin texture in momentum space^15^,^16^. Advances in angle-resolved photoemission spectroscopy (ARPES) have facilitated to observe these textures in spin-resolved spectra^17^,^18^,^19^,^20^,^21^,^22^. The two routes to break the TRS and to gap the surface state of a 3D TI are either the doping with transition-metal ions as magnetic impurities^23^,^24^ or the magnetic proximity effect of a magnetic insulator (MI) adlayer or substrate^25^,^26^. In magnetically doped TIs, Dirac semi-metallic surface states acquire a gap and reveal a hedgehog-like spin texture^23^; their Hall conductance is quantized in units of *e*^2^/*h*^5^,^6^,^27^.

The isostructural tetradymite compounds Bi_2_Se_3_, Bi_2_Te_3_ and Sb_2_Te_3_ belong to the class of strong TIs with an odd number of massless Dirac cones at selected surfaces^15^,^28^. Bi_2_Se_3_ has a band gap of 0.3 eV and only one massless Dirac cone in the surface-band dispersion, if the crystal is cleaved along the (111) direction^29^,^30^. Ab initio GW calculations have challenged the results of earlier band structure calculations and concluded that the band gap is direct^31^. Experimentally, ARPES^19^,^31^,^32^,^33^,^34^ or scanning tunneling microscopy^35^ leave this issue still unsettled. Typically, Se vacancies at the surface shift the Fermi level towards the conduction band^36^, but further doping by Ca counteracts this shift and can move the Fermi level back to the Dirac point^37^. The real-space structure of Bi_2_Se_3_ consists of stacked layers. In this stacking, Bi and Se alternate to form five-layer blocks which are coupled via van der Waals interactions; it is therefore well suited for preparing thin films or heterostructures. A structure of five such layers, typically referred to as the ‘quintuple’ layer, repeats along the (111) direction^38^,^39^.

Here, we focus on a slab geometry for a 3D TI, in which the exchange field acts on only one of the surface layers. This choice naturally applies to a geometry, in which a TI slab is attached to a ferromagnetic insulator. We adopt a previously-developed strategy to describe a slab of Bi_2_Se_3_, stacked with *N*_*z*_ quintuple layers along the *z*-direction^40^. The formalism, as outlined in the Method section, straightforwardly allows to examine the layer-resolved electronic dispersion of the slab with respect to the transverse momenta. The low-energy bands of Bi_2_Se_3_ result from four bonding and anti-bonding *P*_*z*_ orbitals with total angular momenta *J*_*z*_ = ±1/2. Below, we will refer to the *J*_*z*_ eigenvalues in short as “spin”. If one of its surfaces is exposed to a magnetic field or exchange coupled to a ferromagnetic insulator, such a slab has the same hedgehog spin-texture in momentum space as in magnetically doped TIs. However, at a critical field strength, the hedgehog texture transforms into a skyrmion texture. This topological transition is signalled by a discrete change in the skyrmion counting number. It originates from a field-induced degeneracy point of a surface and a bulk band which, thereafter, interchange their spatial characters. Remarkably, the spin-texture transition leaves the Hall conductance *σ*_*xy*_ = *e*^2^/2*h* unchanged. The skyrmion “spin” texture remains stable over a finite range of exchange fields similar to the real-space skyrmion lattices in chiral magnets in an external magnetic field^41^.

## Results

### “Spin” texture

**Figure 1:**
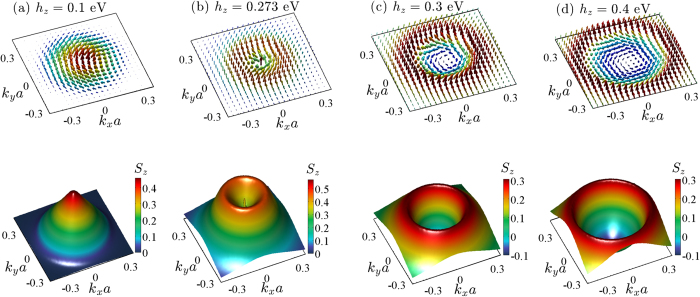
Hedgehog and skyrmion “spin” textures. “Spin” texture **S**(**k**) (top row) and the *z*-component of the “spin” expectation value *S*_*z*_(**k**) (in units of *ħ*/2) (bottom row) in the vicinity of the Dirac point at the surface Brillouin zone center 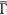 on the exchange-coupled surface of the Bi_2_Se_3_ slab. (**a**) The texture is hedgehog-like at the field value *h*_*z*_ = 0.1 eV. (**c**) At *h*_*z*_ = 0.3 eV, the texture is skyrmion-like. (**b**) The texture at *h*_*z*_ = 0.273 eV is close to the topological transition. (**d**) At *h*_*z*_ = 0.4 eV, the texture has lost its skyrmion structure. The values for the critical exchange fields are determined from the evaluation of the skyrmion counting number (see text and Fig. 3(a)). *S*_*z*_ at the 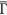 point changes discontinuously from a positive to a negative value upon crossing the critical exchange field at which the transition from a hedgehog to a skyrmion texture takes place.

**Figure 2:**
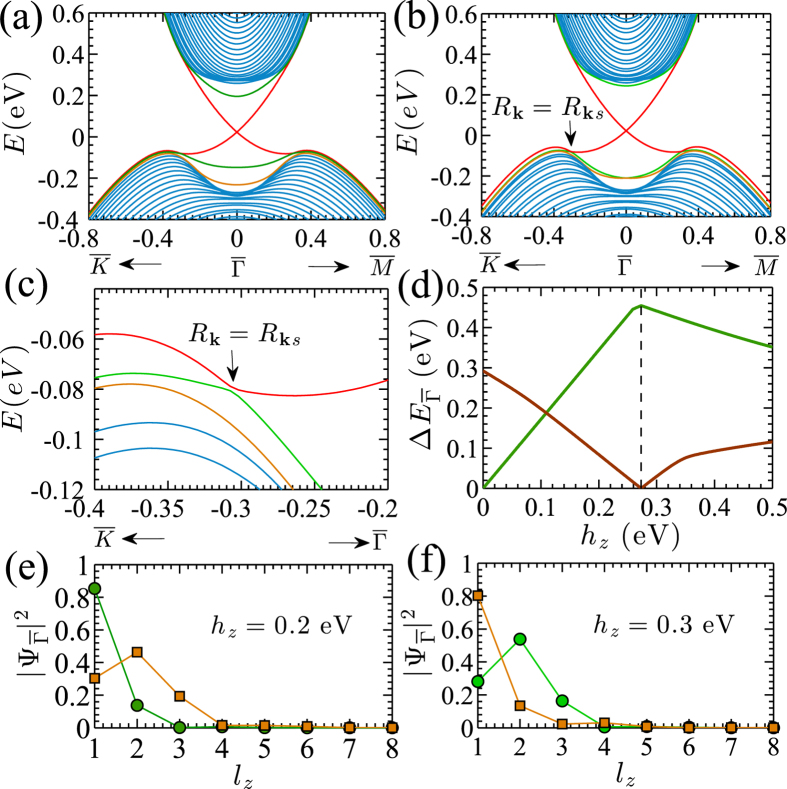
Electronic structure across the topological transition. (**a**,**b**) Band dispersions of the Bi_2_Se_3_ slab near the 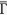 point. The unsplit and exchange-split surface bands are marked in red and green, respectively, for field strengths (**a**) *h*_*z*_ = 0.2 eV (hedgehog phase), and (**b**) *h*_*z*_ = *h*_*zc*1_ = 0.273 eV at the transition. The arrow indicates the special radius 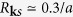 for the avoided level crossing of the two-surface bands. (**c**) Expanded view of the spectrum in (**b**) near *R*_**k**_ = *R*_**k***s*_. (**d**) The energy gaps 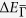 at the 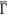 point of the exchange-split surface band (green) and between the occupied part of this band and the top occupied bulk band (brown). The squared amplitude of the wave function 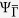 for the green and orange bands in (**a**,**b**) as a function of the layer index *l*_*z*_ for (**e**) *h*_*z*_ = 0.2 eV (hedgehog phase) and (**f**) *h*_*z*_ = 0.3 eV (skyrmion phase).

In the absence of a magnetic or an exchange field, two degenerate Dirac cones appear in the spectrum near the center of the surface Brillouin zone, the 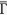 point; the corresponding states are spatially confined to the top or the bottom surface. With the Bi_2_Se_3_ specific parameter set adopted from ref. 40, the Dirac point is not precisely located at the Fermi energy, but this has no influence on the results presented below. Once the TRS is broken by a finite field of strength *h*_*z*_ in one of the two surfaces of the slab, the two-fold degeneracy is lifted in all the bands and the surface state, which experiences the exchange field, acquires a gap. In Fig. 1, we plot the momentum-space “spin” texture **S**(**k**) and the *z*-component of the “spin” expectation value *S*_*z*_(**k**) projected into the surface layer (enumerated as *l*_*z*_ = 1), which is subject to the exchange field, in the vicinity of the Dirac point in the 2D surface Brillouin zone. **S**(**k**) is evaluated as the sum of the *l*_*z*_ = 1 contributions from the two surface-centered bands, top and bottom (marked in red and green in Fig. 2(a,b)). The resultant of the two bands is taken here, because the surface bands hybridize away from the Brillouin zone center (see below and the Supplementary Information). The “spin” texture in the selected surface layer changes qualitatively upon increasing the exchange field. The texture in Fig. 1(a) for *h*_*z*_ = 0.1 eV is “hedgehog”-like. A similar pattern was detected in the spin-resolved ARPES experiments on Mn doped Bi_2_Se_3_^23^. For larger field strength, the momentum-space “spin” structure transforms into a skyrmion-like texture as shown in Fig. 1(c). Most noticeable is the sign change of *S*_*z*_ in the near vicinity of the surface Brillouin zone center, the 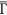 point (|**k**| = 0). Increasing *h*_*z*_ further leads to yet another qualitative change of the “spin” texture. At first sight, the texture in Fig. 1(d) appears to have changed only quantitatively in comparison with Fig. 1(c). But as the analysis below will reveal, the topological character of these textures is indeed qualitatively different.

### Skyrmion number

**Figure 3:**
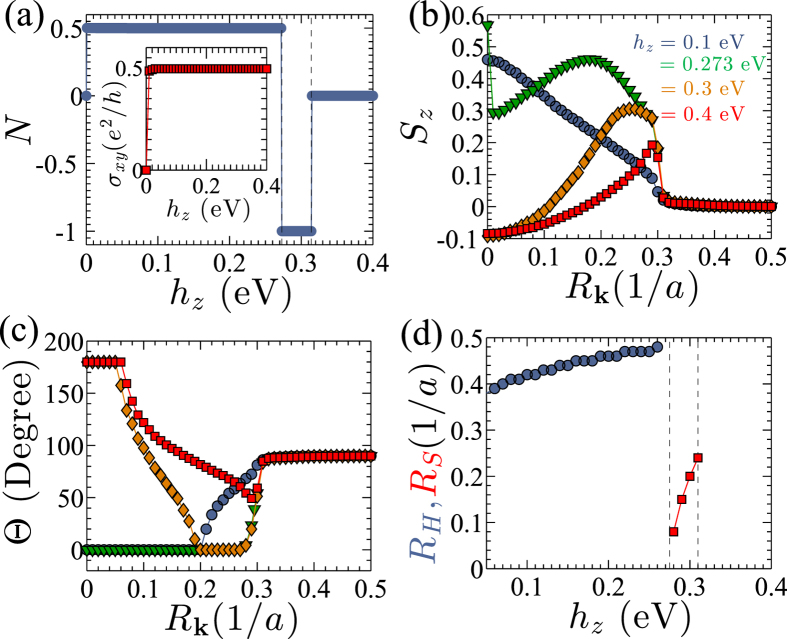
Analysis of the “spin” textures. (**a**) The skyrmion number *N* as a function of the exchange-field strength *h*_*z*_. The dashed vertical lines at the critical fields *h*_*zc*1_ and *h*_*zc*2_ bound the field range in which the skyrmion “spin” texture appears. Inset: Hall conductance 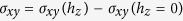 versus *h*_*z*_. 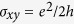 throughout the finite-field range. (**b**) The *z*-component of the “spin” expectation value *S*_*z*_ (in units of *ħ*/2) and (**c**) the polar angle 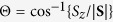 versus the distance 
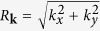
 from the 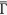 point for different values of *h*_*z*_. The symbols and colors used in (**c**) refer to the same parameters as in (**b**). (**d**) The variation of the characteristic radii *R*_*H*_ (blue circles) and *R*_*S*_ (red squares) of the hedgehog and the skyrmion “spin” textures, respectively.

In order to decisively identify the topological character of the “spin” textures in Fig. 1, we calculate the skyrmion number 
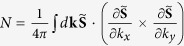
 in the exchange-split occupied surface band (the green band in Fig. 2(c)) at the top surface (*l*_*z*_ = 1) of the slab, where the integral is extended to the hexagonal surface Brillouin zone. 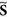 is the normalized “spin” expectation value which ensures the quantization of the skyrmion number. *N* as a function of the exchange field strength *h*_*z*_ is shown in Fig. 3(a). Indeed, *N* = 1/2 for exchange fields below the critical value *h*_*zc*1_ = 0.273 eV, identifying more precisely that the hedgehog phase has the “spin” texture of a half-skyrmion (or meron). At *h*_*zc*1_, the skyrmion number switches to −1, indicating the (anti)-skyrmion character of the texture for 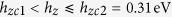, and *N* = 0 beyond *h*_*zc*2_. The discontinuous changes of *N* decisively display the signals for topological phase transitions. *N* takes a finite value (1/2 or −1) in the exchange-split surface band (green band in Fig. 2(a–c)) only and is zero in the unsplit surface band (red band in Fig. 2(a–c)). *N* changes sign upon reversal of the magnetic-field direction. Two types of skyrmion lattices commonly appear in chiral magnets. They are either classified as Néel-type or Bloch-type skyrmion (see *e*.*g*. refs 42, 43, 44); both have the same skyrmion number, but they differ in their spin-winding pattern. A closer inspection of Fig. 1(a) reveals that the momentum-space texture emerging here is a Bloch-type skyrmion.

### Hall conductance

The obvious question arises whether the topological “spin” texture transitions are accompanied by a change in the Chern number and the associated Hall conductance. To address this question, we calculate *σ*_*xy*_ for the full slab via the Kubo formula^45^


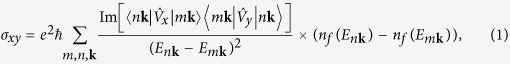


where *m* and *n* are the band indices, 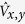 are the velocity operators and *n*_*f*_ denotes the Fermi-Dirac distribution function. The energy gap in thin slabs of 3D TIs is not truly closed at the Dirac point due to a finite size effect even in the absence of a TRS breaking magnetic field^32^,^40^,^46^,^47^,^48^,^49^. *σ*_*xy*_ takes a finite value even for *h*_*z*_ = 0 due to the tiny energy gap at the 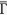 point. Therefore, to isolate the effect of the TRS breaking exchange field, we evaluate and plot 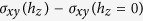 in the inset of Fig. 3(a). The dependence of 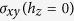 on the number of layers is discussed in the Supplementary Information. As expected for our current set up, which is equivalent to an interface between a 3D TI slab and a ferromagnetic insulator, *σ*_*xy*_ takes the quantized half-integer value *e*^2^/2*h*^14^. *σ*_*xy*_ changes its sign when the magnetic-field direction is reversed^8^. Remarkably, *σ*_*xy*_ does not change at the critical exchange fields, at which the topological “spin” texture transitions take place. We thus encounter the unusual example for topological phase transitions without an energy gap-closing at the Fermi level and without a change in the Chern number. Examples for the former aspect have been presented in ref. 50.

### Characteristic radii

The characteristic “spin” texture in the exchange-split surface band in the surface layer with finite *h*_*z*_ is particularly evident within a circular region around the 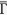 point. Characteristic momentum-space radii *R*_*H*_ and *R*_*S*_ can be determined at which the polar angle 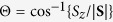 of the “spins” has changed by 90° or 180° for the hedgehog and the skyrmion pattern, respectively, upon moving radially outward from the 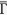 point. Figure 3(b) shows the variation of *S*_*z*_ in the occupied part of the exchange-split surface band with respect to 
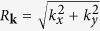
 and thereby identifies the special radius 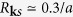 inside which the characteristic hedgehog and skyrmion textures form. At *R*_**k***s*_, |**S**(**k**)| sharply drops to nearly zero. **S**(**k**) in the unsplit surface band has a complementary pattern beyond *R*_**k***s*_ (see Supplementary Information, Fig. S1). As discussed above (see also Fig. 3(b)), 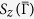 changes sign at the critical field *h*_*zc*1_.

As illustrated in Fig. 3(c), the polar angle 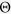, calculated in the occupied part of the exchange-split surface band, continuously varies from 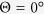 at *R*_**k**_ = 0 to 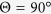 at *R*_**k**_ = *R*_*H*_ for the hedgehog texture, and from 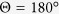 at *R*_**k**_ = 0 to 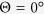 at *R*_**k**_ = *R*_*S*_ for the skyrmion texture. The plateaus, appearing at 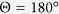 and 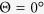 for the skyrmion-“spin” texture, establish a distinctive difference to the typical spatial structure of skyrmions in chiral magnets^44^. With increasing *h*_*z*_, the characteristic radius *R*_*H*_ for the hedgehog texture increases slowly within the field range 0 < *h*_*z*_ < *h*_*zc*1_, while the radius *R*_*S*_ for the skyrmion texture increases rapidly within the field range 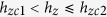 as shown in Fig. 3(d). *R*_*H*_ and *R*_*S*_ even exceed further out than the special radius *R*_**k***s*_. Beyond *h*_*zc*2_, 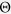 stops at a finite angle and the “spins” no longer sweep to the opposite direction indicating the loss of the texture’s skyrmion character.

### Electronic spectra across the transition

To get more insight into the origin of the topological phase transition, we analyze the changes in the electronic structure across the transition. In Figs 2(a,b), the band dispersions of the slab are plotted in the hedgehog phase (*h*_*z*_ = 0.2 eV) and at the critical field *h*_*zc*1_ = 0.273 eV, respectively, along the 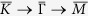 direction in the hexagonal surface Brillouin zone. Upon increasing *h*_*z*_, the top occupied bulk band (orange) rises up in energy and touches the exchange-split surface band (green) at the 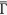 point for *h*_*z*_ = *h*_*zc*1_, as depicted in Fig. 2(b). The former turns back towards the lower-energy bulk bands upon further increasing *h*_*z*_.

The exchange-split and unsplit surface bands have an avoided level crossing at 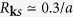, as visible in Fig. 2(c). This observation clarifies the role of the special radius *R*_**k***s*_ within which the hedgehog and skyrmion textures form. The hybridization between the two (top and bottom) surface bands of the slab is possible, because their corresponding wave functions extend towards the interior of the slab at momenta away from the 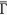 point and therefore allow for a finite overlap (see also the Supplementary Information).

Figure 2(d) shows the variation of the energy gap at the 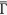 point of the exchange-split surface band and the gap between the occupied part of this band and the top occupied bulk band. When the exchange field reaches *h*_*z*_ = *h*_*zc*1_, a bulk and a surface states become degenerate at the 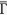 point. Figure 2(e,f) show the squared amplitude of the wave functions at the 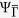, calculated for the occupied exchange-split surface band and the top occupied bulk band, as a function of the layer index *l*_*z*_ for *h*_*z*_ = 0.2 eV (hedgehog phase) and *h*_*z*_ = 0.3 eV (skyrmion phase). Evidently, these states interchange their spatial character across the transition.

## Discussion

An experimental detection of the skyrmion texture will be challenging using spin-resolved ARPES techniques. The real obstacle, however, to induce the topological transition is the required large exchange splitting. For the Bi_2_Se_3_ specific parameter set which we have used in our calculations, the required exchange field is more than four times larger than the so far observed splitting of ~50 meV in Bi_2_Se_3_ samples which are homogeneously doped with magnetic impurities^24^. At the TI/MI heterointerface of Bi_2_Se_3_/MnSe(111), the exchange splitting is only 7 meV^51^,^52^. Yet, the extraordinarily large g-factor of ~50 observed for the Dirac electrons in the Bi_2_Se_3_ surface states may render it possible to achieve unusually large exchange splittings^25^,^53^. We have verified that the critical field can be reduced by applying an electric field along *z*-direction (up to ~15% by a bias voltage of 0.1 V between the two open surfaces). The phenomenon of the topological transition is expected to be generic to other strong TIs as well. Therefore, the selection of a TI with a band gap, narrower than Bi_2_Se_3_, is another possible route to realize the anticipated topological transition or the “spin”-skyrmion texture in momentum space itself. Explicit calculations confirm the expectation that temperature effects are negligibly small for the observed phase transition because of the material’s sizeable energy gap of 0.3 eV. Hence, the transitions should robustly occur at room temperature and even beyond. For these temperatures, orbital effects arising from the magnetization of the surface will not be relevant, justifying a posteriori the ansatz that the exchange field couples only to the electron’s spin. Furthermore, the typical cyclotron frequencies *ω*_*c*_ in semiconductors are of the order *ω*_*c*_ ~ 10^11^ × H[Tesla] Hz. Specifically, for Bi_2_Se_3_, an inverse scattering rate *τ*_*s*_ ~ 5.1 × 10^−14^ s was inferred from de-Haas-van Alphen experiments^54^. So *ω*_*c*_*τ*_*s*_ < 1 even for magnetic fields near 100 T, indicating that the effects of orbital magnetic-field are unlikely to influence the surface electrons in Bi_2_Se_3_.

The encountered topological phase transition provides a new example where the energy gap at the Fermi level does not close across the transition. Remarkably, while the skyrmion counting number changes, the Hall conductance remains constant. The hedgehog to skyrmion phase transition in the momentum-space “spin” texture is yet another striking phenomenon to occur in three dimensional topological insulators.

## Method

The Hamiltonian for a slab of Bi_2_Se_3_ is given by [ref. 40, Supplementary Information]


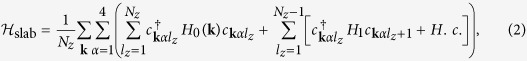


where 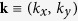, index *α* labels the four bonding and antibonding states of *P*_*z*_ orbitals in the following order: 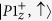, 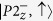, 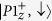, 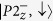; these orbitals form the low-energy bands of Bi_2_Se_3_. The superscripts denote the parity^28^, *l*_*z*_ is the layer index, and the arrows represent the total angular momentum eigenvalues *J*_*z*_ = ±1/2 which result from spin-orbit coupling^22^.

A single quintuple layer, in the presence of a perpendicular exchange (or Zeeman) field, is effectively described by the Hamiltonian^15^


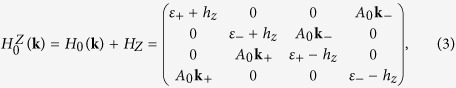


with 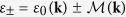, 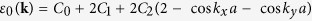, 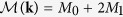 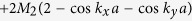, 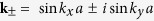, *a* is the lattice constant in a layer, *h*_*z*_ is the strength of the exchange field, and *H*_*z*_ describes the exchange coupling via the *h*_*z*_ entries on the matrix diagonal. *H*_1_ accounts for the coupling between two neighboring layers and is expressed as


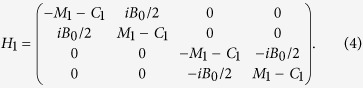


The parameters in *H*_0_ and *H*_1_ are taken from ref. 40: *A*_0_ = 0.8 eV, *B*_0_ = 0.32 eV, *C*_0_ = −0.0083 eV, *C*_1_ = 0.024 eV, *C*_2_ = 1.77 eV, *M*_0_ = −0.28 eV, *M*_1_ = 0.216 eV, *M*_2_ = 2.6 eV and *a* = 4.14 Å.

The exchange field is subsequently chosen to act only on the top surface layer of the slab with layer index *l*_*z*_ = 1. The total Hamiltonian matrix for the slab, of dimension 4*N*_*z*_ × 4*N*_*z*_, therefore, has the tridiagonal structure


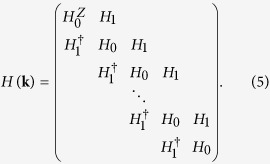


The band dispersion *E*_**k**_ of the slab is obtained by solving the eigenvalue equation 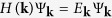, where 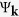 and *E*_**k**_ are the eigenvectors and eigenvalues of *H*(**k**), respectively. The “spin” expectation values, at the surface layer with exchange coupling, are computed using


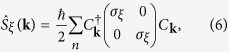


where  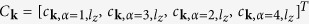 with *l*_*z*_ = 1, *n* labels the eigenenergies corresponding to the two surface bands, 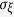 (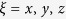) are the Pauli matrices, and *ħ* is the Planck’s constant. The results presented above are obtained for a slab of 15 quintuple layers.

## Additional Information

**How to cite this article**: Mohanta, N. *et al*. Emergent Momentum-Space Skyrmion Texture on the Surface of Topological Insulators. *Sci. Rep.* **7**, 45664; doi: 10.1038/srep45664 (2017).

**Publisher's note:** Springer Nature remains neutral with regard to jurisdictional claims in published maps and institutional affiliations.

## Supplementary Material

Supplementary Information

## References

[b1] Fu L., Kane C. L. & Mele E. J. Topological insulators in three dimensions. Phys. Rev. Lett. 98, 106803, doi: 10.1103/PhysRevLett.98.106803 (2007). 17358555

[b2] Fu L. & Kane C. L. Topological insulators with inversion symmetry. Phys. Rev. B 76, 045302, doi: 10.1103/PhysRevB.76.045302 (2007).

[b3] Hasan M. Z. & Kane C. L. *Colloquium*: Topological insulators. Rev. Mod. Phys. 82, 3045–3067, doi: 10.1103/RevModPhys.82.3045 (2010).

[b4] Moore J. E. The birth of topological insulators. Nature (London) 464, 194–198, doi: 10.1038/nature08916 (2010). 20220837

[b5] Yu R. . Quantized anomalous Hall effect in magnetic topological insulators. Science 329, 61–64, doi: 10.1126/science.1187485 (2010). 20522741

[b6] Chang C.-Z. . Experimental observation of the quantum anomalous Hall effect in a magnetic topological insulator. Science 340, 167–170, doi: 10.1126/science.1234414 (2013). 23493424

[b7] Kou X. . Scale-invariant quantum anomalous Hall effect in magnetic topological insulators beyond the two-dimensional limit. Phys. Rev. Lett. 113, 137201, doi: 10.1103/PhysRevLett.113.137201 (2014). 25302915

[b8] Wang J., Lian B. & Zhang S.-C. Quantum anomalous Hall effect in magnetic topological insulators. Physica Scripta 2015, 014003, doi: 10.1088/0031-8949/2015/T164/014003 (2015).

[b9] Jiang H., Qiao Z., Liu H. & Niu Q. Quantum anomalous Hall effect with tunable Chern number in magnetic topological insulator film. Phys. Rev. B 85, 045445, doi: 10.1103/PhysRevB.85.045445 (2012).

[b10] Duong L. Q., Lin H., Tsai W.-F. & Feng Y. P. Quantum anomalous Hall effect with field-tunable Chern number near *Z*_2_ topological critical point. Phys. Rev. B 92, 115205, doi: 10.1103/PhysRevB.92.115205 (2015).

[b11] Garate I. & Franz M. Inverse spin-galvanic effect in the interface between a topological insulator and a ferromagnet. Phys. Rev. Lett. 104, 146802, doi: 10.1103/PhysRevLett.104.146802 (2010). 20481953

[b12] Li R., Wang J., Qi X.-L. & Zhang S.-C. Dynamical axion field in topological magnetic insulators. Nat. Phys. 6, 284–288, doi: 10.1038/nphys1534 (2010).

[b13] Essin A. M., Moore J. E. & Vanderbilt D. Magnetoelectric polarizability and axion electrodynamics in crystalline insulators. Phys. Rev. Lett. 102, 146805, doi: 10.1103/PhysRevLett.102.146805 (2009). 19392469

[b14] Qi X.-L., Hughes T. L. & Zhang S.-C. Topological field theory of time-reversal invariant insulators. Phys. Rev. B 78, 195424, doi: 10.1103/PhysRevB.78.195424 (2008).

[b15] Zhang H. . Topological insulators in Bi_2_Se_3_, Bi_2_Te_3_ and Sb_2_Te_3_ with a single Dirac cone on the surface. Nat. Phys. 5, 438–442, doi: 10.1038/nphys1270 (2009).

[b16] Yazyev O. V., Moore J. E. & Louie S. G. Spin polarization and transport of surface states in the topological insulators Bi_2_Se_3_ and Bi_2_Te_3_ from first principles. Phys. Rev. Lett. 105, 266806, doi: 10.1103/PhysRevLett.105.266806 (2010). 21231702

[b17] Hsieh D. . Observation of unconventional quantum spin textures in topological insulators. Science 323, 919–922, doi: 10.1126/science.1167733 (2009). 19213915

[b18] Souma S. . Direct measurement of the out-of-plane spin texture in the Dirac-cone surface state of a topological insulator. Phys. Rev. Lett. 106, 216803, doi: 10.1103/PhysRevLett.106.216803 (2011). 21699328

[b19] Pan Z.-H. . Electronic structure of the topological insulator Bi_2_Se_3_ using angle-resolved photoemission spectroscopy: Evidence for a nearly full surface spin polarization. Phys. Rev. Lett. 106, 257004, doi: 10.1103/PhysRevLett.106.257004 (2011). 21770666

[b20] Jozwiak C. . Widespread spin polarization effects in photoemission from topological insulators. Phys. Rev. B 84, 165113, doi: 10.1103/PhysRevB.84.165113 (2011).

[b21] Qi X.-L. & Zhang S.-C. Topological insulators and superconductors. Rev. Mod. Phys. 83, 1057–1110, doi: 10.1103/RevModPhys.83.1057 (2011).

[b22] Zhang H., Liu C.-X. & Zhang S.-C. Spin-orbital texture in topological insulators. Phys. Rev. Lett. 111, 066801, doi: 10.1103/PhysRevLett.111.066801 (2013). 23971598

[b23] Xu S.-Y. . Hedgehog spin texture and Berry’s phase tuning in a magnetic topological insulator. Nat. Phys. 8, 616–622, doi: 10.1038/nphys2351 (2012).

[b24] Chen Y. L. . Massive Dirac fermion on the surface of a magnetically doped topological insulator. Science 329, 659–662, doi: 10.1126/science.1189924 (2010). 20689013

[b25] Wei P. . Exchange-coupling-induced symmetry breaking in topological insulators. Phys. Rev. Lett. 110, 186807, doi: 10.1103/PhysRevLett.110.186807 (2013). 23683236

[b26] Lang M. . Proximity induced high-temperature magnetic order in topological insulator - ferrimagnetic insulator heterostructure. Nano Letters 14, 3459–3465, doi: 10.1021/nl500973k (2014). 24844837

[b27] Bestwick A. J. . Precise quantization of the anomalous Hall effect near zero magnetic field. Phys. Rev. Lett. 114, 187201, doi: 10.1103/PhysRevLett.114.187201 (2015). 26001016

[b28] Liu C.-X. . Model Hamiltonian for topological insulators. Phys. Rev. B 82, 045122, doi: 10.1103/PhysRevB.82.045122 (2010).

[b29] Larson P. . Electronic structure of Bi_2_X_3_ (*x* = S, Se, T) compounds: Comparison of theoretical calculations with photoemission studies. Phys. Rev. B 65, 085108, doi: 10.1103/PhysRevB.65.085108 (2002).

[b30] Pertsova A. & Canali C. M. Probing the wavefunction of the surface states in Bi_2_Se_3_ topological insulator: a realistic tight-binding approach. New Journal of Physics 16, 063022, doi: 10.1088/1367-2630/16/6/063022 (2014).

[b31] Nechaev I. A. . Evidence for a direct band gap in the topological insulator Bi_2_Se_3_ from theory and experiment. Phys. Rev. B 87, 121111, doi: 10.1103/PhysRevB.87.121111 (2013).

[b32] Zhang Y. . Crossover of the three-dimensional topological insulator Bi_2_Se_3_ to the two-dimensional limit. Nat. Phys. 6, 584–588, doi: 10.1038/nphys1689 (2010).

[b33] Sánchez-Barriga J. . Photoemission of Bi_2_Se_3_ with circularly polarized light: Probe of spin polarization or means for spin manipulation? Phys. Rev. X 4, 011046, doi: 10.1103/PhysRevX.4.011046 (2014).

[b34] Neupane M. . Observation of quantum-tunnelling-modulated spin texture in ultrathin topological insulator Bi_2_Se_3_ films. Nat. Commun. 5, 3841, doi: 10.1038/ncomms4841 (2014). 24815418

[b35] Kim S. . Surface scattering via bulk continuum states in the 3D topological insulator Bi_2_Se_3_. Phys. Rev. Lett. 107, 056803, doi: 10.1103/PhysRevLett.107.056803 (2011). 21867088

[b36] Xia Y. . Observation of a large-gap topological-insulator class with a single Dirac cone on the surface. Nat. Phys. 5, 398–402, doi: 10.1038/nphys1274 (2009).

[b37] Hsieh D. . A tunable topological insulator in the spin helical Dirac transport regime. Nature (London) 460, 1101–1105, doi: 10.1038/nature08234 (2009). 19620959

[b38] Cava R. J., Ji H., Fuccillo M. K., Gibson Q. D. & Hor Y. S. Crystal structure and chemistry of topological insulators. J. Mater. Chem. C 1, 3176–3189, doi: 10.1039/C3TC30186A (2013).

[b39] Wang L.-L. & Johnson D. D. Ternary tetradymite compounds as topological insulators. Phys. Rev. B 83, 241309, doi: 10.1103/PhysRevB.83.241309 (2011).

[b40] Ebihara K., Yada K., Yamakage A. & Tanaka Y. Finite size effects of the surface states in a lattice model of topological insulator. Physica E 44, 885–890, doi: 10.1016/j.physe.2011.12.008 (2012).

[b41] Bauer A. & Pfleiderer C. Magnetic phase diagram of MnSi inferred from magnetization and ac susceptibility. Phys. Rev. B 85, 214418, doi: 10.1103/PhysRevB.85.214418 (2012).

[b42] Roszler U. K., Bogdanov A. N. & Pfleiderer C. Spontaneous skyrmion ground states in magnetic metals. Nature (London) 442, 797–801, doi: 10.1038/nature05056 (2006). 16915285

[b43] Kezsmarki I. . Néel-type skyrmion lattice with confined orientation in the polar magnetic semiconductor GaV_4_S_8_. Nat. Mater. 14, 1116–1122, doi: 10.1038/nmat4402 (2015). 26343913

[b44] Nagaosa N. & Tokura Y. Topological properties and dynamics of magnetic skyrmions. Nat. Nano. 8, 899–911, doi: 10.1038/nnano.2013.243 (2013). 24302027

[b45] Kohmoto M. Topological invariant and the quantization of the Hall conductance. Annals of Physics 160, 343–354, doi: 10.1016/0003-4916(85)90148-4 (1985).

[b46] Linder J., Yokoyama T. & Sudbø A. Anomalous finite size effects on surface states in the topological insulator Bi_2_Se_3_. Phys. Rev. B 80, 205401, doi: 10.1103/PhysRevB.80.205401 (2009).

[b47] Sakamoto Y., Hirahara T., Miyazaki H., Kimura S.-i. & Hasegawa S. Spectroscopic evidence of a topological quantum phase transition in ultrathin Bi_2_Se_3_ films. Phys. Rev. B 81, 165432, doi: 10.1103/PhysRevB.81.165432 (2010).

[b48] Liu C.-X. . Oscillatory crossover from two-dimensional to three-dimensional topological insulators. Phys. Rev. B 81, 041307, doi: 10.1103/PhysRevB.81.041307 (2010).

[b49] Ozawa H., Yamakage A., Sato M. & Tanaka Y. Topological phase transition in a topological crystalline insulator induced by finite-size effects. Phys. Rev. B 90, 045309, doi: 10.1103/PhysRevB.90.045309 (2014).

[b50] Ezawa M., Tanaka Y. & Nagaosa N. Topological phase transition without gap closing. Scientific Reports 3, 2790, doi: 10.1038/srep02790 (2013). 24071900 PMC3784957

[b51] Eremeev S. V., Men’shov V. N., Tugushev V. V., Echenique P. M. & Chulkov E. V. Magnetic proximity effect at the three-dimensional topological insulator/magnetic insulator interface. Phys. Rev. B 88, 144430, doi: 10.1103/PhysRevB.88.144430 (2013).

[b52] Li M. . Proximity-driven enhanced magnetic order at ferromagnetic-insulator21magnetic-topological-insulator interface. Phys. Rev. Lett. 115, 087201, doi: 10.1103/PhysRevLett.115.087201 (2015). 26340203

[b53] Analytis J. G. . Two-dimensional surface state in the quantum limit of a topological insulator. Nat. Phys. 6, 960–964, doi: 10.1038/nphys1861 (2010).

[b54] Lawson B. J., Hor Y. S. & Li L. Quantum oscillations in the topological superconductor candidate Cu_0.25_Bi_2_S_3_. Phys. Rev. Lett. 109, 226406, doi: 10.1103/PhysRevLett.109.226406 (2012). 23368142

