# Supplementary material for: Emergent Momentum-Space Skyrmion Texture on the Surface of Topological Insulators
Source: Sci Rep. 2017 Apr 5;7:45664. doi: 10.1038/srep45664 (PMC5381213; doi:10.1038/srep45664)
Supplement: Supplementary Information [file srep45664-s1.pdf]

Supplementary Information: Emergent Momentum-Space Skyrmion Texture on the Surface of Topological Insulators

Narayan Mohanta[1,*], Arno P. Kampf[1], and Thilo Kopp[2]
Center for Electronic Correlations and Magnetism,
[1]*Theoretical Physics III,* [2]*Experimental Physics VI,*
Institute of Physics, University of Augsburg, 86135 Augsburg, Germany

*Correspondence to narayan.mohanta@physik.uni-augsburg.de

S1. Derivation of the Hamiltonian for a slab geometry

The Hamiltonian for a slab geometry of Bi_2Se_3 was derived earlier in Ref. [1] (Ref. [32] in the main text) and the derivation is briefly revisited below. Open boundary conditions are imposed along the (111) direction (taken to be along the z-axis) while periodic boundary conditions are used for the perpendicular directions. We start with the effective low-energy Hamiltonian matrix for bulk Bi_2Se_3

$$\mathcal{H}_{\rm bulk}(\mathbf{k_3}) = \begin{pmatrix} \bar{\epsilon}_+(\mathbf{k_3}) & \bar{B}_0 k_z & 0 & \bar{A}_0 \bar{k}_- \\ \bar{B}_0 k_z & \bar{\epsilon}_-(\mathbf{k_3}) & \bar{A}_0 \bar{k}_- & 0 \\ 0 & \bar{A}_0 \bar{k}_+ & \bar{\epsilon}_+(\mathbf{k_3}) & -\bar{B}_0 k_z \\ \bar{A}_0 \bar{k}_+ & 0 & -\bar{B}_0 k_z & \bar{\epsilon}_-(\mathbf{k_3}) \end{pmatrix} \tag{S1}$$

which was worked out in Ref. [2] for the orbital basis functions given in the main text. Here, $\mathbf{k_3} \equiv (k_x, k_y, k_z)$, $\bar{\epsilon}_\pm(\mathbf{k_3}) = \bar{\epsilon}_0(\mathbf{k_3}) \pm \bar{\mathcal{M}}(\mathbf{k_3})$, $\bar{k}_\pm = k_x \pm i k_y$, $\bar{\epsilon}_0(\mathbf{k_3}) = \bar{C}_0 + \bar{C}_1 k_z^2 + \bar{C}_2 k_\perp^2$, $\bar{\mathcal{M}}(\mathbf{k_3}) = \bar{M}_0 + \bar{M}_1 k_z^2 + \bar{M}_2 k_\perp^2$. The momenta along and perpendicular to the (111) direction are denoted by k_z and $k_\perp = \sqrt{k_x^2 + k_y^2}$, respectively. The lattice generalization of the above Hamiltonian is obtained by substituting $k_i a_i \approx \sin k_i a_i$ and $(k_i a_i)^2 \approx 2(1 - \cos k_i a_i)$ leading to

$$\mathcal{H}_{\rm lattice}(\mathbf{k_3}) = \begin{pmatrix} \tilde{\epsilon}_+(\mathbf{k_3}) & B_0 \sin k_z c & 0 & A_0 k_- \\ B_0 \sin k_z c & \tilde{\epsilon}_-(\mathbf{k_3}) & A_0 k_- & 0 \\ 0 & A_0 k_+ & \tilde{\epsilon}_+(\mathbf{k_3}) & -B_0 \sin k_z c \\ A_0 k_+ & 0 & -B_0 \sin k_z c & \tilde{\epsilon}_-(\mathbf{k_3}) \end{pmatrix} \tag{S2}$$

where $\tilde{\epsilon}_\pm(\mathbf{k_3}) = \tilde{\epsilon}_0(\mathbf{k_3}) \pm \tilde{\mathcal{M}}(\mathbf{k_3})$, $k_\pm = \sin k_x a \pm i \sin k_y a$, $\tilde{\epsilon}_0(\mathbf{k_3}) = C_0 + 2C_1(1 - \cos k_z c) + 2C_2(2 - \cos k_x a - \cos k_y a)$, $\tilde{\mathcal{M}}(\mathbf{k_3}) = M_0 + 2M_1(1 - \cos k_z c) + 2M_2(2 - \cos k_x a - \cos k_y a)$. The parameters are modified by powers of the lattice constants a and c, *i.e.* $A_0 = \bar{A}_0/a$, $B_0 = \bar{B}_0/c$, $C_0 = \bar{C}_0$, $C_1 = \bar{C}_1/c^2$, $C_2 = \bar{C}_2/a^2$, $M_0 = \bar{M}_0$, $M_1 = \bar{M}_1/c^2$, $M_2 = \bar{M}_2/a^2$.

The finite thickness of the slab is accounted for by performing the partial reverse Fourier transformation of the fermionic operators $c_{\mathbf{k_3}} = (1/N_z) \sum_{l_z} e^{ik_z l_z} c_{k_x,k_y,l_z}$. The Hamiltonian for the slab then reads

$$\begin{aligned} \mathcal{H}_{\rm slab} &= \sum_{\mathbf{k_3}} c^\dagger_{\mathbf{k_3}} \mathcal{H}_{\rm lattice}(\mathbf{k_3}) c_{\mathbf{k_3}} \\ &= \frac{1}{N_z^2} \sum_{\mathbf{k_3}} \sum_{l_z, l'_z} e^{-ik_z l_z} c^\dagger_{k_x,k_y,l_z} \mathcal{H}_{\rm lattice}(\mathbf{k_3}) e^{ik_z l'_z} c_{k_x,k_y,l'_z}. \end{aligned} \tag{S3}$$

With the exponential forms for the sine and cosine functions and the identities $\sum_{k_z} e^{ik_z(l_z - l'_z - 1)} = N_z \delta_{l_z, l'_z+1}$, $\sum_{k_z} e^{ik_z(l_z - l'_z + 1)} = N_z \delta_{l_z, l'_z-1}$, the Hamiltonian (S3) reduces to the Hamiltonian (1) in the main text.

S2. Complementary "spin" textures

The hedgehog and skyrmion textures appear mainly in the occupied part of the exchange split surface states. A more precise statement is hampered by the fact that the two surface bands hybridize around the avoided level-crossing momenta $R_{\mathbf{k}s} = \sqrt{k_x^2 + k_y^2} \simeq 0.3/a$. Near the $\bar{\Gamma}$ point, the surface states are truly confined to either the top

or the bottom surface layer. Yet, upon moving away from the $\bar{\Gamma}$ point, the surface states spatially extend continuously more towards the interior of the slab. The concomitant increasing overlap of the surface states' wave functions in the central layer of the slab is the origin of the hybridization of the exchange split and the unsplit band which are truly surface bands only at and near the $\bar{\Gamma}$ point. For momenta beyond $R_{\mathbf{k}s}$, the spatial character of the surface bands is interchanged (see Fig. 3(c) in the main text).

The hybridization and the avoided level crossing at $R_{\mathbf{k}s}$ result in a complementarity of the associated "spin" textures. This is demonstrated in Fig. S1. This figure shows the "spin" texture of both surface bands within the surface layer in which the exchange field is applied. For the exchange-split surface band (the green band in Fig. 3(a), (b), (c) in the main text), the "spin" expectation values sharply drop to nearly zero when the magnitude of the momentum exceeds the ring with radius $R_{\mathbf{k}s}$. The origin of this drop is the significantly reduced amplitude of the wave function in the selected surface layer due to the interchange of the surface states and their associated spatial character at the avoided level crossing momenta with magnitude $R_{\mathbf{k}s}$.

The "spin" expectation values for the unsplit surface band (the red band in Fig. 3(a), (b), (c) in the main text) display the complementary behavior. Within the selected surface layer, the "spin" expectation values drop to nearly zero when the magnitude of the momenta is smaller than the ring radius $R_{\mathbf{k}s}$ – for the same reason as outlined above, *i.e.* the interchange of the character of the two surface bands at $R_{\mathbf{k}s}$.

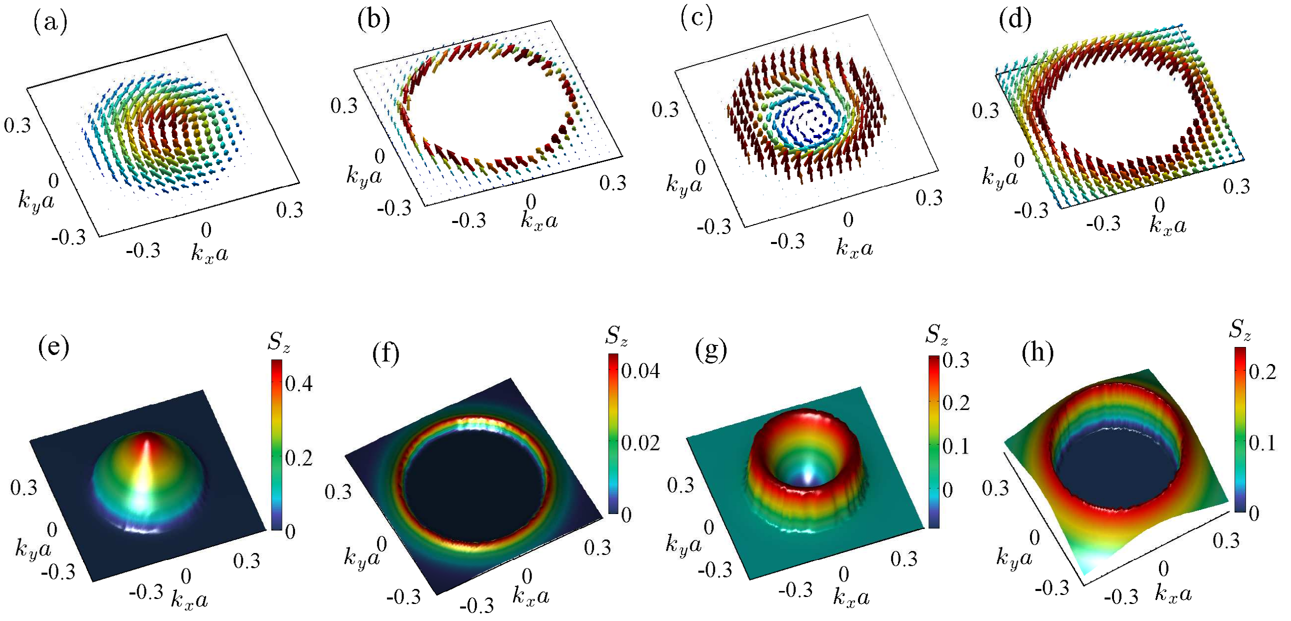

FIG. S1. **Complementary "spin" textures in the surface bands.** "Spin" texture of the surface bands in the surface layer which is subject to the exchange field: $h_z = 0.1$ eV (hedgehog phase) in (a), (b) and (e), (f); $h_z = 0.3$ eV (skyrmion phase) in (c), (d) and (g), (h). (b) and (d) show the textures in the occupied highest-energy band (the red band in Fig. 3(a), (b), (c)). At and near the $\bar{\Gamma}$ point, the eigenstates of this band are spatially confined in the surface layer in which no field is applied. (a) and (c) show the corresponding textures in the second-to-highest energy band (the green band in Fig. 3(a), (b), (c)). Figures (e), (f), (g), and (h) show selectively S_z only (in units of $\hbar/2$).

S3. Finite-size effect on the Hall conductance

As studied previously in Ref. [3–7], the energy gap in thin slabs of three dimensional topological insulator is not truly closed at the Dirac point even in the absence of time-reversal symmetry breaking magnetic field. This gap decreases in an oscillatory manner with increasing the layer number N_z. As discussed in Ref. [3–7], there is a phase transition from a band insulator to a topologically non-trivial insulating phase at $N_z = 3$, made evident by an increase in the energy gap at the $\bar{\Gamma}$ point as shown in Fig. S2. More transitions follow with increasing N_z involving parity changes of the bands closest to the Fermi level [6]. The oscillations in the energy gap with increasing N_z are shown in the inset of Fig. S2. A non-zero energy gap prevails for all slab thicknesses even though it shrinks to tiny values with increasing N_z. This energy gap causes a finite contribution to σ_{xy} even in the absence of any exchange field. As shown in Fig. S2, σ_{xy} increases linearly with N_z beyond $N_z = 4$. The variation of σ_{xy} with N_z is connected to the Berry

curvature of the surface-band states. The Berry curvature is largest near the $\bar{\Gamma}$ point and increases with decreasing energy gap. The rise in the energy gap at $N_z = 3$ is also reflected as a cusp in σ_{xy}. σ_{xy} is calculated for the full slab, and, therefore, it is the conductance rather than the conductivity. This explains the linear rise of σ_{xy} with the slab thickness for $n_z > 4$.

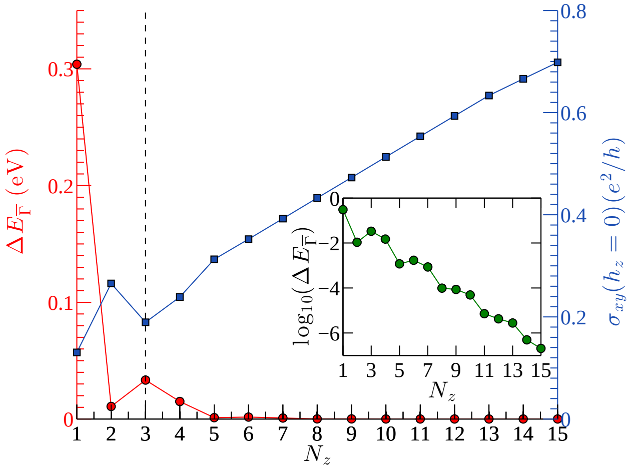

FIG. S2. **Finite-size effect and Hall conductance at zero field.** The variation of the energy gap $\Delta E_{\bar{\Gamma}}$ at the $\bar{\Gamma}$ point (vertical scale on the left) and the Hall conductance σ_{xy} in the absence of an exchange field (vertical scale on the right) with respect to the layer number N_z. The conductance σ_{xy} is calculated for the full slab, and, therefore, approaches a linear thickness dependence for $N_z > 4$. Inset: $\Delta E_{\bar{\Gamma}}$ on the logarithmic scale as a function of N_z. The dashed line indicates a critical layer thickness ($N_z = 3$) at which a transition takes place from a band insulator to a topological insulator.

REFERENCES

[1] Ebihara, K. *et al.* Finite size effects of the surface states in a lattice model of topological insulator. *Physica E* **44**, 885 (2012).

[2] Liu, C.-X. *et al.* Model Hamiltonian for topological insulators. *Phys. Rev. B* **82**, 045122 (2010).

[3] Linder, J., Yokoyama, T. & Sudbø, A. Anomalous finite size effects on surface states in the topological insulator Bi_2Se_3. *Phys. Rev. B* **80**, 205401 (2009).

[4] Zhang, Y. *et al.* Crossover of the three-dimensional topological insulator Bi_2Se_3 to the two-dimensional limit. *Nature Phys.* **6**, 584 (2010).

[5] Sakamoto, Y. *et al.* Spectroscopic evidence of a topological quantum phase transition in ultrathin Bi_2Se_3 films. *Phys. Rev. B* **81**, 165432 (2010).

[6] Liu, C.-X. *et al.* Oscillatory crossover from two-dimensional to three-dimensional topological insulators. *Phys. Rev. B* **81**, 041307 (2010).

[7] Ozawa, H., Yamakage, A., Sato, M. & Tanaka, Y. Topological phase transition in a topological crystalline insulator induced by finite-size effects. *Phys. Rev. B* **90**, 045309 (2014).
